# Untargeted Metabolomics Analysis Reveals Potential Metabolic Targets in Gemcitabine-Treated Pancreatic Cancer Cells

**DOI:** 10.3390/metabo16070471

**Published:** 2026-07-06

**Authors:** Arjun Prasad Tiwari, Blake R. Rushing, Larissa Silva, Susan J. Sumner, Pinku Mukherjee

**Affiliations:** 1 Department of Biological Sciences, University of North Carolina at Charlotte, Charlotte, NC 28223, USA; atiwari5@charlotte.edu (A.P.T.); lsilva@charlotte.edu (L.S.); 2 Department of Nutrition, UNC Chapel Hill, Kannapolis, NC 28010, USA; blake_rushing@unc.edu; 3 Nutrition Research Institute, UNC Chapel Hill, Kannapolis, NC 28010, USA

**Keywords:** metabolites, pancreatic ductal adenocarcinoma, untargeted metabolomics, pathway analysis, N-acetylneuraminic acid

## Abstract

Background/Objectives: Pancreatic ductal adenocarcinoma (PDAC) is a highly aggressive malignancy characterized by limited treatment options and poor prognosis. Gemcitabine is a commonly used chemotherapy; however, gemcitabine resistance in PDAC poses a critical barrier to effective treatment, as the underlying mechanisms are not yet fully understood. Methods: This study employs an exploratory untargeted metabolomics approach to investigate metabolic differences in PDAC cells in the presence and absence of gemcitabine treatment. HPAF-II, MIA PaCa-2, and BxPC-3 cell lines were used as models for gemcitabine-resistant, moderately responsive, and permissive PDAC cells, respectively. Results: MTT assay results revealed that BxPC-3 cells are highly sensitive to gemcitabine treatment, HPAF-II cells are the most resistant, and MIA PaCa-2 cells exhibit moderate sensitivity. Orthogonal Partial Least Squares Discriminant Analysis (OPLS-DA) of the metabolomics data demonstrated clear differentiation of gemcitabine-treated and untreated (control) cells. When comparing the treated vs. control conditions, 170 metabolites matched to an in-house library of standards were significant (*p* < 0.05 or fold change ≥ 2 or VIP ≥ 1) differentiators in HPAF-II cells, whereas MIA PaCa-2 and BxPC-3 cells had 178 and 218 differentiating metabolites, respectively. HPAF-II cells treated with gemcitabine had significantly higher levels of N-acetylneuraminic acid and 7-dehydrocholesterol compared with the control group. In contrast, these metabolites were significantly lower or non-significant in BxPC-3 treated cells. Pathway analysis revealed that the steroid biosynthesis pathway was significantly perturbed in HPAF-II cells, whereas amino sugar and nucleotide sugar metabolism was predominantly altered in BxPC-3 cells. Conclusions: Overall, this exploratory study reveals metabolic differences between treated and untreated cells to derive targeted therapeutic strategies that could be used in the future to improve treatment outcomes for PDAC patients.

## 1. Introduction

Pancreatic ductal adenocarcinoma (PDAC) is characterized by poor prognosis and low survival rates, due to late-stage diagnosis, aggressive tissue invasion, and limited treatment options [1]. Chemotherapy is the primary treatment for pancreatic cancer, due to its potent cytotoxic potential, accessibility, and affordability. Gemcitabine is a widely used chemotherapeutic agent, administered either alone or in combination with other drugs and therapies [2]. However, resistance to gemcitabine often develops rapidly, leading to treatment failure or relapse shortly after dose completion [3]. Despite extensive research, the underlying mechanisms of this resistance remain largely unknown. Previous studies suggest that altered drug metabolism, enhanced repair of DNA damage, the repopulation of cancer stem-like cells, and the evasion of apoptosis are key contributors to chemoresistance [4,5,6,7]. Conventional molecular research approaches have primarily emphasized gene and protein markers associated with chemoresistance, [8,9] but are inherently limited, and do not capture a broad spectrum of molecular changes in response to treatment. The role of metabolic alterations related to gemcitabine resistance in PDAC remains largely underexplored, although some studies have shown evidence that metabolic variation in PDAC cells may be linked to therapy response [10,11]. Previous studies using Seahorse assays have demonstrated metabolic differences among PDAC cell lines, particularly in lactate and pyruvate levels as well as alterations in glycolytic gene expression [12]. Gebregiworgis et al. reported that gemcitabine-resistant cells exhibit a metabolic shift away from glycolysis compared with gemcitabine-sensitive cells [13]. This metabolic reprogramming facilitates the diversion of glucose-derived carbon into nucleotide biosynthesis pathways, leading to the accumulation of endogenous nucleotides. The increased nucleotide pool functions as a competitive gemcitabine inhibitor, thereby contributing to gemcitabine resistance. These observations underscore the importance of metabolic adaptations in gemcitabine response and provide a foundation for comparative metabolomic analyses across pancreatic cancer cell lines with varying degrees of gemcitabine sensitivity. Metabolomics has emerged as a powerful tool for profiling a broad range of metabolite classes and determining corresponding pathway perturbations in response to a wide variety of stressors in biological systems. Untargeted metabolomics enables the identification and annotation of tens of thousands of metabolites, including previously uncharacterized compounds [14]. This comprehensive coverage provides deeper insights into molecular mechanisms underlying therapeutic response.

In this exploratory study, we aimed to determine how gemcitabine treatment impacts the metabolic profiles of three PDAC cell types. To our knowledge, this is the first study using metabolomics to analyze gemcitabine-treated PDAC cell lines with varying degrees of gemcitabine sensitivity: highly resistant, moderately responsive, and highly sensitive. Our findings reveal that each PDAC subtype responds differently to gemcitabine treatment, as evidenced by distinct metabolic perturbations. Due to the exploratory nature of this study, further investigations are needed to validate these findings using additional methods and model systems.

## 2. Materials and Methods

### 2.1. Cell Culture

Human PDAC cell lines (HPAF-II, MIA PaCa-2, and BxPC-3) were obtained from the American Type Culture Collection. HPAF-II (ATCC CRL-1997) carries mutations in CDKN2A, KRAS, and TP53. MIA PaCa-2 (ATCC CRM-CRL-1420) harbors mutations in KRAS and TP53. BxPC-3 (ATCC CRL-1687) contains mutations in CDKN2A, MAP2K4, SMAD4, and TP53. Cells were cultured in MEM, DMEM and RPMI 1640 media for HPAF-II, MIA PaCa-2, and BxPC-3 cells, respectively, supplemented with 1% penicillin/streptomycin (Gibco, Charlotte, NC, USA), 1% GlutaMAX (Gibco, Charlotte, NC, USA), 10% fetal bovine serum (Gibco, Charlotte, NC, USA), and 1% non-essential amino acids (Cellgro, Charlotte, NC, USA). Gemcitabine hydrochloride (hereafter, Gemcitabine) (Catalog No. S1149, Purity: 99.96%) was purchased from Selleckchem, Charlotte, NC, USA. N-acteylmannosamine (Sigma-Aldrich, A8176, Charlotte, NC, USA) was purchased from Sigma Aldrich. Distilled water was used to dissolve the gemcitabine at a concentration of 100 mM as a stock. This stock solution was further diluted in culture medium to prepare the working concentration as necessary. Since the original solvent for gemcitabine was water, no solvent-related effects on the cells were expected. Therefore, a vehicle control group was not included. Control cells refer to cells that were left completely untreated.

### 2.2. MTT Assay

The MTT (3-[4,5-dimethylthiazol-2-yl]-2,5-diphenyl tetrazolium bromide) assay was performed following the manufacturer’s instructions. Cells were seeded at a density of 5000 cells per well in a 96-well plate and incubated for 48 h. Cells were then treated with various concentrations of gemcitabine (0.1 µM to 10 µM) for an additional 24 h. After treatment, MTT reagent was added and incubated for 3 h. Formazan crystals were dissolved by adding dimethyl sulfoxide, and absorbance was measured at 540 nm using an ELISA reader (ThermoFisher, Charlotte, NC, USA). Untreated cells served as the control group, representing 100% cell viability. Similarly, cells were treated with a combination of gemcitabine and N-acetylmannosamine (ManNAc), and cell viability was assessed. Double-distilled water was used to dissolve ManNAc powder to prepare a 100 mM stock solution and further diluted in respective culture medium serially during treatment. To determine the optimal concentration of ManNAc, a concentration-response analysis was performed across a range of 1 to 1000 µM. Based on these results, 100 µM ManNAc was selected for subsequent co-treatment experiments with gemcitabine.

### 2.3. Clonogenic Assay

For clonogenic analysis, cells (500 per well) were seeded in 12-well plates for 24 h. Cells were then treated with gemcitabine and ManNAc, alone or in combination, and incubated for 7 days to permit colony formation. Cell colonies were fixed with methanol:acetic acid (3:1) and stained with 0.5% crystal violet [15]. After washing and air-drying, colonies were visualized using the ChemiDoc MP Imaging System (Bio-Rad, Hercules, CA, USA). Later, Image J (ImageJ 1.54g, NIH, Bethesda, MD, USA) was used to quantify the colonies as performed by others with some modifications [16]. Clonogenic survival is expressed as a surviving fraction using the following formula:

$$Surviving\;fraction=\frac{Colonies\;counted\;after\;treatment}{Cells\;seeded\;\times \;plating\;efficiency}$$

where plating efficiency is calculated using

$$plating\;efficiency=\frac{Number\;of\;colonies\;formed\times 100\;}{Number\;of\;cells\;seeded\;}$$

Statistical significance was defined as *p* < 0.05.

### 2.4. Sample Preparation for Metabolomic Profiling

Untargeted metabolomics was conducted on cellular extracts using ultra-high-performance liquid chromatography–high-resolution mass spectrometry (UHPLC-HRMS) according to previous methods [17,18]. Briefly, 2 × 10^6^ cells were cultured on 10 cm plates under standard conditions (37 °C, 5% CO_2_) until reaching 70–80% confluence. Notably, cells were passed one day prior to the treatment ensuring that the cells were all in the log phase of the cell cycle. Cells were treated with 1 µM gemcitabine for 48 h in their respective culture media. Following treatment, cells were washed twice with 10 mL of ice-cold phosphate-buffered saline (PBS, 1X, pH 7.4). Cells were quenched by the addition of −20 °C acetonitrile (1 mL), which was rolled around the plate to ensure uniform coverage. An additional 750 µL of ice-cold water was added to the acetonitrile-quenched plates, and cells were thoroughly scraped and collected into 15 mL conical tubes. This extraction process was repeated, and both extracts for each plate were pooled, giving a total extract volume of 3.5 mL. Samples were vortexed for 10 min and centrifuged at 16,000× *g* for 10 min at 4 °C. The supernatant was transferred to new cryovials and the pellets were used for measuring protein concentrations. Supernatants were dried overnight by SpeedVac and reconstituted in 95:5 water:methanol in a volume proportional to each sample’s protein concentration. Metabolic profiling was conducted using four independent samples from the same group.

### 2.5. Untargeted Metabolomics Data Capture, Data Processing, and Metabolite Identification/Annotation

UHPLC-HRMS analysis was performed using a Vanquish UHPLC coupled to a Q-Exactive HFx Orbitrap mass spectrometer [19]. Chromatographic separation of metabolites was conducted on a Waters Acquity C18 column (2.1 mm × 100 mm, 1.7 µm particle size). The mobile phase consisted of (A) 0.1% formic acid in water and (B) 0.1% formic acid in methanol. The solvent gradient was initially held at 1% B for 1 min, increased to 99% B over 15 min, and then held at 99% B for 4 min. A flow rate of 400 µL/min was used throughout the LC method. Mass spectral data was collected from 70 to 1050 *m*/*z* in positive mode, and a data-dependent acquisition method was used to fragment the top 20 most abundant ions. Blank and Quality Control Study Pool (QCSP) injections were interspersed evenly throughout the analysis run. Raw data were preprocessed using Progenesis QI. Background was removed by filtering out signals with a higher average abundance in the blank samples compared to the QCSP samples. Data were normalized using the “normalize to all” feature in Progenesis. Metabolite identifications and annotations were made using algorithms that matched signals to an in-house physical standards library of compounds and public databases.

### 2.6. Statistical Analysis and Pathway Analysis of Metabolomics Data

Multivariate analysis was performed on the normalized UHPLC-HRMS data using MetaboAnalyst 6.0 to reduce dimensionality and visualize separation among phenotypic groups [20]. Unsupervised principal component analysis (PCA) models were generated and examined to confirm that QCSP samples clustered tightly and were in the center of the study samples from which they were derived, a commonly used quality control measure in metabolomics [21]. Supervised orthogonal partial least squares discriminant analysis (OPLS-DA) was applied to examine separation of study groups and identify variables with high importance for group separation [22]. Similarly, partial least squares discriminant analysis (PLS-DA) was also performed for further confirmation of separation of study groups.

Univariate statistical analyses were performed in MetaboAnalyst 6.0 using two-sided *t*-tests. Metabolites were reported as differentiating the groups if they met at least one of the following criteria: variable importance in projection (VIP)  ≥  1.0, fold change ≥  2, *p*  <  0.05. False discovery rate (FDR) correction was not applied due to the exploratory nature of the study [23,24]. Pathway enrichment analyses were conducted with the “Enrichment Analysis” module in MetaboAnalyst using in-house matched metabolites that were significantly altered in each comparison.

### 2.7. Enzyme-Linked Immunosorbent Assay (ELISA) to Measure Endogenous N-Acetylneuraminic Acid

To determine the effect of ManNAc supplementation on intracellular N-acetylneuraminic acid levels, each cell line was treated with gemcitabine alone, ManNAc (100 µM) alone, or a combination of gemcitabine and ManNAc. Following treatment, cells were lysed using RIPA lysis buffer (1X). The following lysates were analyzed using a Human N-acetylneuraminic Acid ELISA Kit (EK711403, Delft, AFG Bioscience, Netherlands) according to the manufacturer’s instructions to quantify intracellular N-acetylneuraminic acid. Briefly, standards and samples were added to the antibody-coated microplate and incubated with the provided detection reagents. Following a series of wash steps, TMB substrate was added for color development, and the reaction was terminated using stop solution. Absorbance was measured at 450 nm using a microplate reader, and N-acetylneuraminic acid concentrations were calculated from the standard curve generated using the supplied standards.

### 2.8. Statistical Analysis of Cell Assay Data

Cell assay data was analyzed using GraphPad Prism 9.0, employing two-way ANOVA multiple comparisons unless otherwise stated. A *p*-value of <0.05 was considered statistically significant. Three independent biological experiments (*n* = 3) were performed. Within each experiment, samples were analyzed in triplicate wells. Data are presented as mean ± standard deviation (SD).

## 3. Results

### 3.1. Differential Sensitivity of PDAC Cell Lines to Gemcitabine Treatment

**Figure 1:**
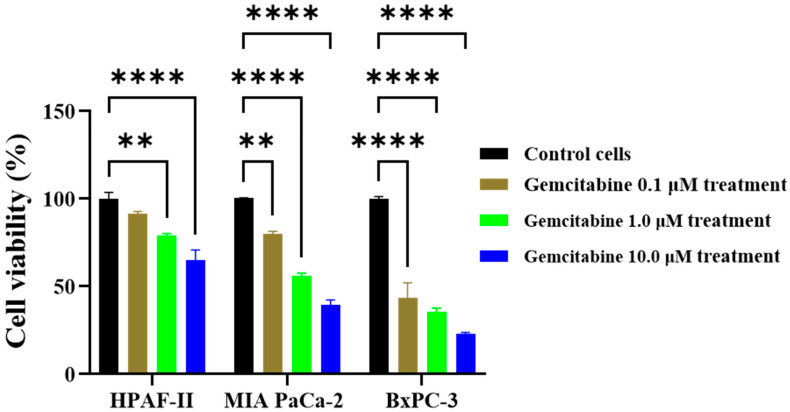
MTT assay of different cell lines post gemcitabine treatment. Statistical significance was conducted by 2-way ANOVA (nonparametric or mixed model, multiple comparison). A *p* value < 0.05 was considered significant (** *p* < 0.01, and **** *p* < 0.0001).

We assessed the gemcitabine response in three cell lines using the MTT assay. Our results in Figure 1 indicate that gemcitabine treatment led to a concentration-dependent decrease in cell viability across all cell lines. However, the degree of sensitivity varied, with HPAF-II as the least sensitive, MIA PaCa-2 as moderately sensitive, and BxPC-3 as the most sensitive (Figure 1). At a 1 µM gemcitabine concentration, cell viability was 79% for HPAF-II, 55% for MIA PaCa-2, and 35% for BxPC-3.

### 3.2. Metabolomics Comparison of PDAC Cells Following Gemcitabine Treatment

**Figure 2:**
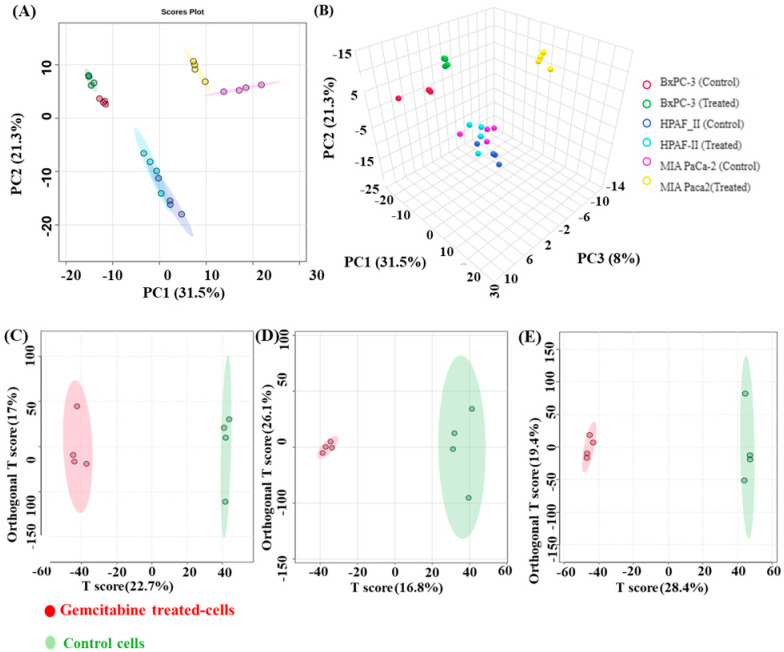
PCA score plot of all cell extracts (**A**) and synchronized three-dimensional score plot (**B**). OPLS-DA plots of gemcitabine-treated cells vs. control cells; HPAF-II (**C**), MIA PaCa-2 (**D**) and BxPC-3 (**E**).

Following data preprocessing and filtering, a total of 10,221 features remained in the metabolomics dataset, with 438 features matched to the in-house physical standards library. Using only in-house matched metabolites, PCA was used to visualize major differences in the metabolomics profiles between the cell types and treatment groups. The PCA score plots demonstrated separation between treated and untreated groups for each of the cell lines (Figure 2A,B). This is further supported by a combined PCA score plot and synchronized three-dimensional score plot (Supplementary Materials, Figures S1A–D). For each cell line, PLS-DA plots showed clear separation between control cells and gemcitabine-treated cells, indicating that gemcitabine treatment substantially alters the overall metabolic profile (Supplementary Materials, Figure S2A–C). Supplementary Materials, Figure S2D, shows a PCA plot of the metabolomics dataset with tight clustering of QC samples in the middle of the study samples following filtering and normalization, which is an indication of high data quality [21].

Supervised OPLS-DA was used to determine metabolites that differentiated (VIP > 1) the control vs treatment groups for each cell type. The OPLS-DA analysis (Figure 2C–E) produced model statistics that overall supported strong separation between treated and control groups: R2X = 0.227, R2Y = 0.837 and Q2 = 0.537 for HPAF-II; R2X = 0.233, R2Y = 0.981, and Q2 = 0.661 for MIA PaCa-2; and R2X = 0.284, R2Y = 0.972 and Q2 = 0.784 for BxPC-3 (Supplementary Materials, Table S1). The observation that R2Y values were greater than 0.5 indicated that gemcitabine treatment significantly altered the metabolome of each cell line [19]. To assess potential model overfitting, permutation testing (100 permutations) was performed for all OPLS-DA models using MetaboAnalyst, and the results are provided in Supplementary Materials, Figure S3. Models for BxPC-3 and MIA PaCa-2 passed permutation testing for both R2Y and Q2 metrics (*p* < 0.05), supporting acceptable model robustness and predictive performance. In contrast, the HPAF-II model passed permutation testing for R2Y but not Q2, suggesting reduced reproducibility and predictive stability of the model which may be due to the higher treatment resistance that we observed for this cell line. To identify the key metabolic perturbations after gemcitabine treatment, a univariate *t*-test (*p* < 0.05, fold change ≥ 2, VIP ≥ 1) was performed using MetaboAnalyst 6.0 using signals that matched to an in-house library of reference standards. The significantly altered metabolites between the gemcitabine-treated cells and control cells are summarized in Supplementary Materials, Table S2, which includes signal details, metabolite names, %CV, *p*-values, fold change and VIP scores. Additionally, a Venn diagram was used to illustrate shared and unique metabolic changes (*p* < 0.05 or fold change ≥ 2 or VIP ≥ 1) among the three cell lines following gemcitabine treatment.

### 3.3. Distinct Metabolic Responses to Gemcitabine Treatment in PDAC Cells

**Figure 3:**
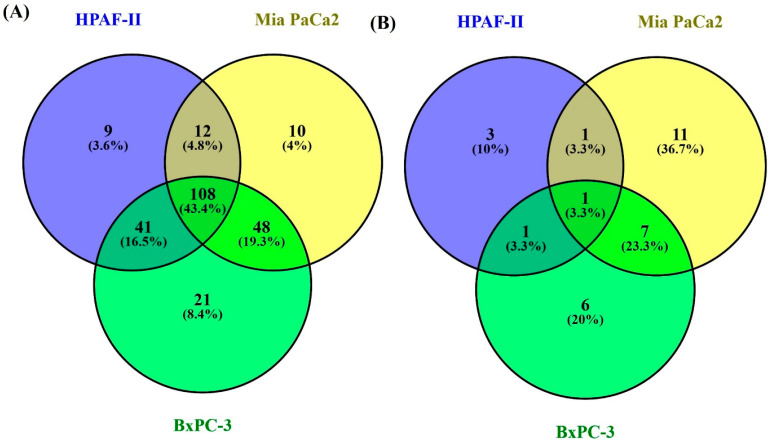
Venn diagrams showing the number and percentages of metabolites (**A**) and pathways (**B**) that were different in gemcitabine-treated vs. controls for the 3 different cell lines. Only metabolites/pathways showing statistically significant differences between gemcitabine-treated and control cells (*p* < 0.05) were included in the Venn diagram.

Metabolic profiling revealed the extent of shared and unique metabolites across PDAC cell lines following gemcitabine treatment. The Venn diagram shows 170 metabolites in HPAF-II cells, 178 in MIA PaCa-2, and 218 in BxPC-3 are altered (Figure 3A). Notably, 108 metabolites were commonly perturbed across all cell lines following gemcitabine treatment including glycerophosphocholine, nicotinamide, N-methylalanine, methylthioadenosine, xanthine, and hypoxanthine, though the magnitude of change varied across different cell types (Supplementary Materials, Table S2—peak statistics). Additionally, 12 metabolites including citric acid, arginine, uric acid, desmosterol and 1-methyladenosine were commonly perturbed between HPAF-II and MIA PaCa-2 cells. Among the commonly perturbed metabolites, the signal for glycerophosphocholine was consistently higher across all cell lines post-treatment, though at varying levels (Supplementary Materials, Figure S4). Increased glycerophosphocholine levels following chemical insults are often linked to apoptosis [25]. Previous reports also showed elevated levels of phosphatidylcholines in gemcitabine-treated tumor-bearing mice [26]. However, the extent of this increase suggests differential cell membrane damage and turnover rates among the cell lines. HPAF-II cells, which are more resistant to gemcitabine, exhibited lower levels of glycerophosphocholine following gemcitabine treatment (*p* < 0.021) compared to control cells. In contrast, MIA PaCa-2 and BxPC-3 cells, which are sensitive to gemcitabine, showed a significant increase in glycerophosphocholine levels compared to untreated cells (*p* < 0.0034), suggesting greater cell membrane damage in both BxPC-3 and MIA PaCa-2 cells. The number of metabolites that met all three criteria (*p* < 0.05 and fold change ≥ 2 and VIP ≥ 1) followed a similar trend across the three cell lines: 73 for HPAF-II, 71 for MIA PaCa-2, and 209 for BxPC-3 (Supplementary Materials, Table S2). Of these, hypoxanthine, xanthine, and methylthioadenosine met this criteria across all three cell lines, indicating that changes in nucleotide metabolism may be some of the most robust metabolic changes following gemcitabine treatment. Further pathway enrichment analysis of the treatment vs control groups for each cell line identified six metabolic pathways significantly altered in HPAF-II cells (Figure 3B, Table 1).

### 3.4. Cell-Type-Specific Metabolic Pathway Alterations Following Gemcitabine Treatment

**Figure 4:**
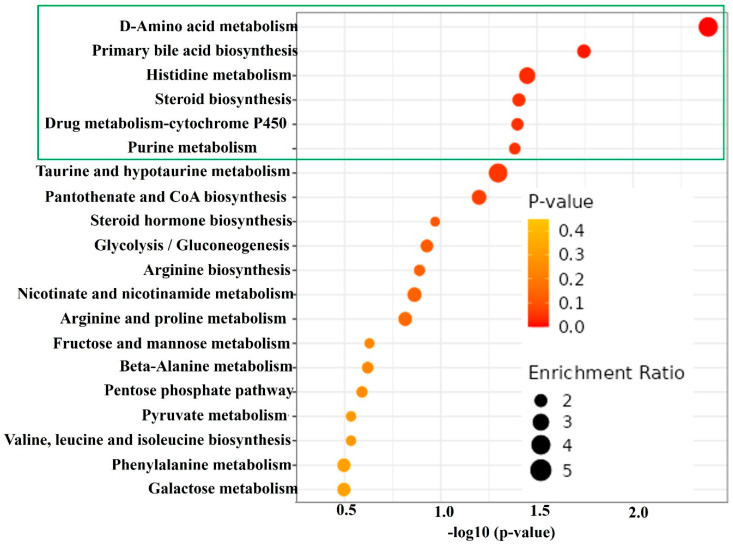
Pathway enrichment analysis of HPAF-II cells. Pathways with *p* < 0.05 are highlighted in the green box.

Metabolic pathway analysis revealed that gemcitabine treatment significantly disrupted multiple pathways across PDAC cell lines (Figure 4, Supplementary Materials, Figures S5 and S6). Only one pathway, D-amino acid metabolism, was commonly affected in all three cell lines. This finding likely reflects a global perturbation of amino acid homeostasis in response to gemcitabine-induced cellular stress. Gemcitabine can trigger oxidative stress, replication stress, and metabolic reprogramming, which could lead to alterations in amino acid abundance and metabolism [27]. The affected pathways in HPAF-II cells include D-amino acid metabolism, primary bile acid biosynthesis, histidine metabolism, steroid metabolism, purine metabolism, and drug metabolism via cytochrome P450 (Figure 4). HPAF-II cells exhibited three uniquely perturbed pathways: primary bile acid biosynthesis, steroid biosynthesis, and drug metabolism–cytochrome P450. In contrast, histidine metabolism was commonly altered in both HPAF-II and MIA PaCa-2 cells. HPAF-II and BxPC-3 cells shared perturbations in purine metabolism (Table 1).

Additionally, arginine and proline metabolism, along with alanine, aspartate and glutamate metabolism, were commonly affected in MIA PaCa-2 and BxPC-3 cells (Supplementary Materials, Figures S5 and S6 and Table S1). Notably, arginine and proline metabolism was significantly enriched in both MIA PaCa-2 and BxPC-3 cells. Corresponding metabolites including putrescine, gamma-aminobutyric acid, spermidine, N-acetyl putrescine, and glutamic acid were significantly downregulated in BxPC-3 cells (Supplementary Materials, Figures S7 and S8) following gemcitabine treatment. 7-Dehydrocholesterol levels were increased in HPAF-II cells following gemcitabine treatment, whereas no significant changes were observed in the other cell lines (Supplementary Materials, Figure S9 and Supplementary Materials, Table S2—peak statistics).

**Figure 5:**
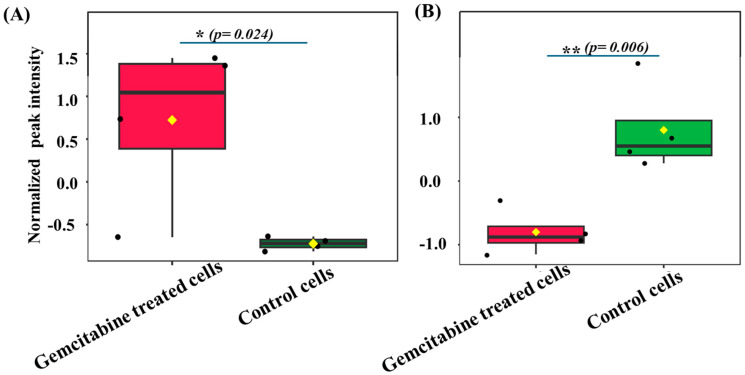
Normalized N-acetylneuraminic acid in HPAF-II (**A**) and BxPC-3 (**B**) cells between gemcitabine treatment and non-treatment groups. Metabolite abundances are scaled to unit variance.

Steroid metabolism was significantly altered only in HPAF-II cells, with no observed effects in MIA PaCa-2 or BxPC-3 cells after gemcitabine treatment. Further analysis identified a notable increase in 7-dehydrocholesterol levels in HPAF-II cells (*p* = 0.007), whereas its expression in BxPC-3 and MIA PaCa-2 cells remained non-significant (*p* > 0.05, Supplementary Materials, Table S2, Peak statistics). Additionally, the amino sugar and nucleotide sugar metabolism pathway was significantly affected in BxPC-3 cells (*p* = 0.007). However, no impact was observed in HPAF-II following gemcitabine treatment (*p* > 0.05). Interestingly, within this pathway, N-acetylneuraminic acid displayed opposing expression patterns across cell types following gemcitabine treatment. Its abundance was elevated markedly in HPAF-II cells (*p* = 0.024 and FC = 3.65) and reduced significantly in BxPC-3 cells (*p* = 0.006) compared to their respective controls (Figure 5A,B). In MIA PaCa-2 cells, levels showed a slight increase (*p* = 0.042), although the fold change was only 1.35 (Supplementary Materials, Figure S10). These findings highlight distinct metabolic responses to gemcitabine treatment.

### 3.5. ManNAc Supplementation Reduces Gemcitabine Sensitivity in PDAC Cells

**Figure 6:**
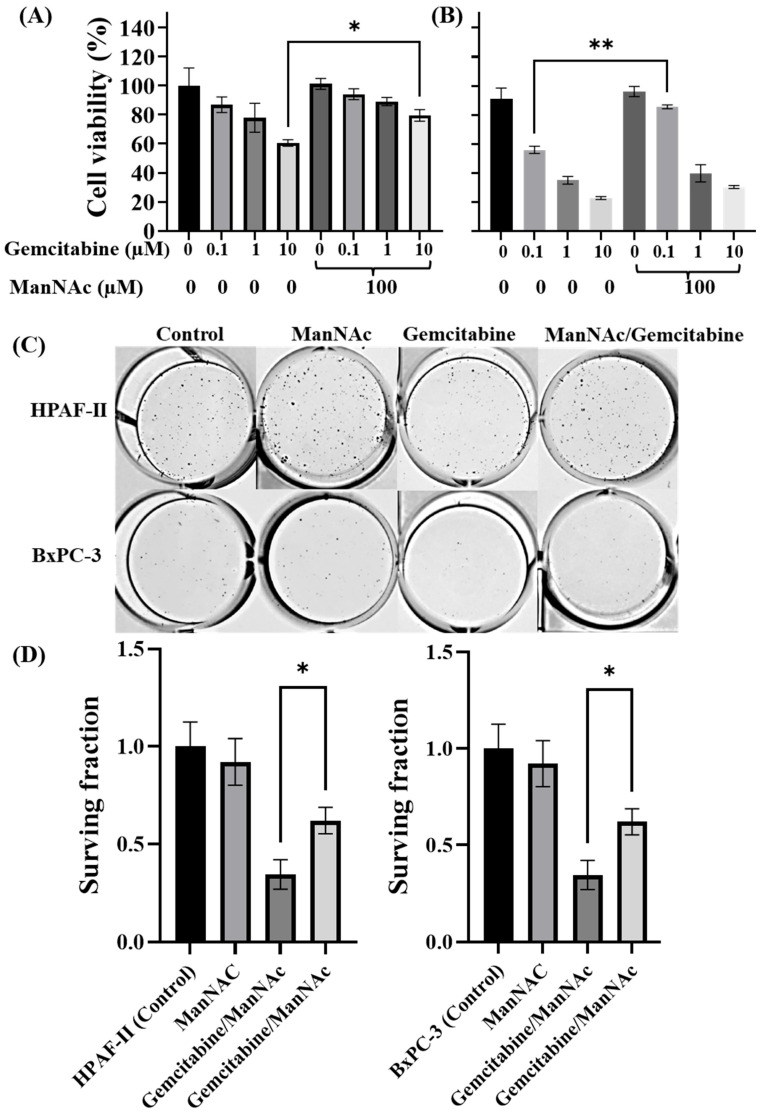
Effects of gemcitabine and ManNAc co-treatments on cell viability and clonogenic survival. MTT assay shows cell viability following co-treatment, HPAF-II (**A**) and BxPC-3 cells (**B**). Colony formation potential (**C**) and surviving efficiency of cells (**D**) after 7 days of indicated treatment. The cells were Co-treated for 48 h for the MTT assay. For the clonogenic assay, 1 µM and 0.1 µM gemcitabine, along with 100 µM ManNAc, were used for HPAF-II and BxPC-3 cells, respectively. A *p* value < 0.05 is considered significant (* *p* < 0.05 and ** *p* < 0.01).

Because HPAF-II cells treated with gemcitabine had significantly higher levels of N-acetylneuraminic acid, HPAF-II and BxPC-3 cells were co-treated with gemcitabine and ManNAc, a membrane-permeable precursor of N-acetylneuraminic acid, to test whether elevated intracellular N-acetylneuraminic acid contributes to reducing gemcitabine sensitivity. Treatment with ManNAc alone had no effect on cell viability (Supplementary Materials, Figure S11), but co-treatment significantly enhanced cell survival depending on the gemcitabine concentration (Figure 6A,B and Supplementary Materials, Figure S12). Co-treatment with 100 µM ManNAc and 10 µM gemcitabine resulted in a HPAF-II cell viability increase to 76% compared to approximately 60% cell viability with gemcitabine alone (Figure 6A). MIA PaCa-2 cells showed protection with ManNAc co-treatment at 0.1 and 1 µM gemcitabine doses (Supplementary Materials, Figure S12), while BxPC-3 cells exhibited significant survival with ManNAc co-treatment at only 0.1 µM gemcitabine (Figure 6B). However, the MIA PaCa-2 and BxPC-3 cells were not rescued by the 100 µM ManNAc co-treatment in presence of 10 µM gemcitabine. The colony formation assay reinforced the cell viability findings in HPAF-II and BxPC-3 cells (Figure 6C). Gemcitabine treatment markedly reduced the surviving fraction to approximately 0.4 in both HPAF-II and BxPC-3 cells. In contrast, supplementation with ManNAc partially restored the surviving fraction to approximately 0.6 in gemcitabine-treated cells (Figure 6D). This result suggests a protective effect of ManNAc against gemcitabine-induced cytotoxicity. However, MIA PaCa-2 cells did not exhibit a colony-forming ability following ManNAc supplementation. Intracellular N-acetylneuraminic acid levels were quantified in HPAF-II, MIA PaCa-2, and BxPC-3 cells following treatment with gemcitabine, ManNAc, or the combination of gemcitabine and ManNAc using ELISA. ManNAc supplementation of a 100 µM significantly increased intracellular N-acetylneuraminic levels in HPAF-II and MIA PaCa-2 cells, whereas no significant increase was observed in BxPC-3 cells (Supplementary Materials, Figure S13). Furthermore, gemcitabine treatment alone induced a significant increase in N-acetylneuraminic acid in HPAF-II cells, while levels were reduced in BxPC-3 cells and unchanged in MIA PaCa-2 cells. The highest N-acetylneuraminic acid levels were observed in HPAF-II and BxPC-3 cells following combination treatment, with levels higher than those with gemcitabine alone.

## 4. Discussion

Gemcitabine is a promising chemotherapeutic agent used either as a monotherapy [28,29] or in combination with other chemotherapeutic drugs such as NAB-paclitaxel, [29] oxaliplatin, [30] and Camptothecin [28] for the treatment of PDAC. However, PDAC is highly refractory to systemic chemotherapy, often leading to limited or transient responses. Although substantial effort has been devoted to uncovering gene-based markers of chemoresistance, [8,9] metabolic rewiring and pathway-level alternations in PDAC cells remain insufficiently explored [31]. In this study, we aimed to investigate the metabolic profile of PDAC cells after gemcitabine treatment, focusing on HPAF-II, MIA PaCa-2, and BxPC-3 cells, which represent varying levels of gemcitabine response: highly resistant, moderately sensitive, and sensitive, respectively. Our cell viability assays confirm that the gemcitabine response in these cell lines is consistent with a previous report [32]. As shown in Table 1, more metabolic pathways are affected in the most sensitive cell line (BxPC-3) than the most resistant cell line (HPAF-II). This aligns with the metabolite alterations observed in these cells, where BxPC-3 cells had a higher number of significantly perturbed metabolites, consistent with their high sensitivity to gemcitabine treatment. Conversely, HPAF-II cells, which are more tolerant to gemcitabine, showed fewer changes in their metabolic profiles. A previous study showed that metabolic trends were completely reversed in resistant cells compared to corresponding wild-type cells [13]. For instance, glycine, taurine, and glycerophospocholine were found reduced in resistant cells relative to wild-type. The most significantly impacted metabolic pathway (as determined by pathway analysis) in BxPC-3 cells was the arginine and proline metabolism pathway. This pathway is critical for maintaining cellular functions such as growth, angiogenesis, and metastasis [33]. The significant downregulation of metabolites in this pathway suggests that gemcitabine treatment impairs downstream cellular processes. In particular, the downregulation of putrescine in BxPC-3 cells (*p* = 0.85 × 10^−4^, VIP = 1.82) indicates a potential dysfunction in these cellular processes. Putrescine is a key molecule involved in cell growth, proliferation, DNA and protein synthesis, and gene regulation [34]. In contrast, HPAF-II cells showed little change in putrescine (*p* = 0.041, VIP = 1.17) and the arginine and proline metabolism pathway was not significantly impacted in the pathway analysis, indicating that HPAF-II cells may retain this functionality despite gemcitabine exposure. Additionally, alanine, aspartate, and glutamate metabolism were significantly affected in BxPC-3 cells (*p* = 0.0012) but not in the other cell lines. The downregulation of L-glutamic acid in BxPC-3 cells suggests alterations in the transamination processes of glutamate and pyruvate, which are critical for generating NADPH in the TCA cycle [35]. A decrease in glutamate may also lead to mitochondrial dysfunction, [36] further contributing to the reduced viability of BxPC-3 cells.

In HPAF-II cells, steroid biosynthesis metabolism was notably perturbed following gemcitabine treatment, with 7-dehydrocholesterol being upregulated (Supplementary Materials, Figure S9). This is in contrast to the other cell lines, where no significant changes were observed (Supplementary Materials, Table S2—peak statistics). Previous studies suggest that 7-dehydrocholesterol may play a role in adaptive responses to chemotherapy. Elevated 7-dehydrocholesterol may suggest activation of alternative survival pathways such as ferroptosis resistance [37,38]. However, the exact mechanism through which 7-dehydrocholesterol contributes to chemoresistance remains unclear and warrants further investigation. Upon further analysis, we observed a subset of metabolites that were commonly altered across all cell types. N-acetylneuraminic acid emerged as a key metabolite with consistent but differential regulation. For example, intracellular N-acetylneuraminic acid levels increased in gemcitabine-resistant HPAF-II cells, whereas decreased levels were observed in gemcitabine-sensitive BxPC-3 cells compared to their respective controls. Previous studies have linked increased sialyltransferase activity, which conjugates N-acetylneuraminic acid, to glycoproteins with gemcitabine resistance [9,39]. Moreover, sialidase-mediated removal of cell surface N-acetylneuraminic acid has been shown to enhance gemcitabine sensitivity in MIA PaCa-2 and BxPC-3 cells [9]. Our findings suggest that the increased intracellular N-acetylneuraminic acid content in HPAF-II cells may play a role in the adaptive response to gemcitabine treatment.

To investigate this hypothesis, we treated HPAF-II and BxPC-3 cells with gemcitabine in combination with ManNAc, a biosynthetic precursor of N-acetylneuraminic acid, and assessed cell viability. This is because polar, uncharged small molecules like ManNAc are more readily taken up by cells than charged molecules such as free N-acetylneuraminic acid [40]. ManNAc was used to elevate intracellular N-acetylneuraminic acid levels [41,42]. Our results further confirm that ManNAc supplementation increases the intracellular N-acetylneuraminic acid pool (Supplementary Materials, Figure S13). We reasoned that if gemcitabine-induced increases in intracellular N-acetylneuraminic acid contribute to cellular adaptation and survival, then supplementing with an additional N-acetylneuraminic acid precursor should further reduce sensitivity of the gemcitabine. Consistent with this idea, HPAF-II cells already relatively resistant to gemcitabine became even more resistant when cultured in ManNAc-supplemented medium compared to ManNAc-free conditions (Figure 6). Other cell lines including BxPC-3 also demonstrated increased cell viability and colony-forming potential when co-treated with gemcitabine. These findings indicate that enhancement of N-acetylneuraminic acid levels through ManNAc supplementation may promote cell survival and contribute to reduced sensitivity to gemcitabine. Supplementation with ManNAc in N-acetylmannosamine kinase-silenced CAPAN cells unable to synthesize endogenous N-acetylneuraminic acid enhanced cell viability and prevented apoptosis [43]. However, the protective mechanism of N-acetylneuraminic acid is not yet fully understood and will be addressed in future studies. Given our lab’s focus on MUC1 signaling, differential expression of N-acetylneuraminic acid across different PDAC cells is particularly relevant. Aberrant hypoglycosylated MUC1 is a hallmark of many cancers [44,45]. Tumor MUC1 is O-glycosylated, with N-acetylneuraminic acid abundantly present as a terminal component [44]. Recent studies have shown MUC1 sialyation in cancer cells directly impacts immune responses through the siglecs-sialylated MUC1 axis [46,47]. Although MUC1 was not directly studied here, the PDAC cells used vary in MUC1 expression (high in HPAF-II, low in MIA PaCa-2, and negligible in BxPC-3) [15]. Therefore, investigating the interrelationship between MUC1 expression and sialylation is essential to determine whether they play regulatory or functionally active roles in modulating each other. Future studies will further investigate the mechanistic basis and functional consequences of the metabolic differences using CRISPR/Cas9 gain-of-function approaches across additional PDAC models. This study highlights the potential of untargeted metabolomics in uncovering metabolites and pathways involved in chemoresistance and increased chemotherapy tolerance in PDAC. Future research should focus on validating these findings and exploring the functional significance of the identified metabolites. A limitation of this study is that multiple-testing correction was not applied to the metabolomics analyses. Given the large number of detected metabolomic features, this increases the possibility of type I errors and false-positive associations. However, the primary objective of this study is exploratory and hypothesis-generating, with the goal of identifying candidate metabolites and pathways. Accordingly, the findings should be confirmed in independent cohorts and through targeted validation studies. Future work incorporating larger sample sizes and orthogonal validation approaches will be necessary to establish the reproducibility and biological significance of our identified metabolites and pathways. Additionally, internal standards were not used to normalize extraction efficiency. Future targeted studies with matched isotope-labeled internal standards should be performed to validate these findings.

## 5. Conclusions

In conclusion, our exploratory study has identified several key metabolites and metabolic perturbations in response to gemcitabine treatment in PDAC cell lines with varying levels of sensitivity. BxPC-3 cells, which exhibited the highest sensitivity to gemcitabine, showed significant changes in 218 metabolites and enriched 15 pathways. In contrast, HPAF-II cells, the most tolerant to gemcitabine, demonstrated fewer changes, with only 170 metabolites and six pathways affected. Pathway enrichment analysis revealed significant alterations in D-amino acid metabolism, primary bile acid biosynthesis, and steroid biosynthesis pathways in HPAF-II treated vs control cells, while notable perturbations in arginine and proline metabolism in treated vs control were found in MIA PaCa-2 and BxPC-3 cells. Our analysis revealed that N-acetylneuraminic acid and 7-dehydrohydrocholesterol levels were significantly increased in the gemcitabine-treated HPAF-II cells compared to corresponding control cells. In contrast, their levels were decreased in BxPC-3 cells. Co-treatment of PDAC cells with ManNAc, an immediate precursor of N-acetylneuraminic acid, reduced their sensitivity to gemcitabine. Therefore, targeting N-acetylneuraminic acid biosynthesis may represent a strategy to resensitize resistant PDAC cells to gemcitabine. Although more validation and mechanistic studies are required, these hypothesis-generating findings lay the foundation for future research aimed at identifying new targets that could be manipulated to enhance chemotherapy sensitivity.

## Figures and Tables

**Table 1:** Significantly perturbed metabolic pathways between control cells and gemcitabine-treated cells.

	*p*-Values		
Metabolic Pathways	HPAF-II	MIA PaCa-2	BxPC-3
Arginine and proline metabolism	ns	1.9 × 10^−7^	2.5 × 10^−5^
Fructose and mannose metabolism	ns	ns	7.5 × 10^−6^
Galactose metabolism	ns	ns	2.9 × 10^−5^
D-Amino acid metabolism	4.0 × 10^−3^	4.7 × 10^−6^	3.0 × 10^−4^
Glycolysis/Gluconeogenesis	ns	ns	6.1 × 10^−4^
Alanine, aspartate and glutamate metabolism	ns	1.1 × 10^−6^	1.2 × 10^−3^
Amino sugar and nucleotide sugar metabolism	ns	4.4 × 10^−2^	2.4 × 10^−3^
Purine metabolism	4.7 × 10^−2^	ns	3.6 × 10^−3^
Inositol phosphate metabolism	ns	ns	8.3 × 10^−3^
Arginine biosynthesis	ns	1.2 × 10^−2^	1.0 × 10^−2^
Neomycin, kanamycin and gentamicin biosynthesis	ns		1.0 × 10^−2^
Pantothenate and CoA biosynthesis	ns	6.0 × 10^−5^	1.0 × 10^−2^
Pyrimidine metabolism	ns	ns	1.4 × 10^−2^
Beta-alanine metabolism	ns	4.0 × 10^−3^	1.5 × 10^−2^
Histidine metabolism	5.0 × 10^−3^	4.0 × 10^−3^	ns
Butanoate metabolism	ns	1.7 × 10^−2^	4.9 × 10^−2^
Nicotinate and nicotinamide metabolism	ns	ns	3.0 × 10^−2^
Glycine, serine and threonine metabolism	ns	4.0 × 10^−5^	4.5 × 10^−2^
Primary bile acid biosynthesis	2.0 × 10^−2^	1.4 × 10^−2^	ns
Steroid biosynthesis	4.4 × 10^−2^	ns	ns
Drug metabolism—cytochrome P450	4.5 × 10^−2^	ns	ns
Phenylalanine metabolism	ns	1.1 × 10^−6^	ns
Tyrosine metabolism	ns	2.4 × 10^−6^	ns
Valine, leucine and isoleucine biosynthesis	ns	6.0 × 10^−5^	ns
Tryptophan metabolism	ns	8.0 × 10^−5^	ns
Glutathione metabolism	ns	7.0 × 10^−3^	ns
Valine, leucine and isoleucine degradation	ns	2.0 × 10^−2^	ns
Cysteine and methionine metabolism	ns	2.9 × 10^−2^	ns
Glyoxylate and dicarboxylate metabolism	ns	4.3 × 10^−2^	ns

## Data Availability

The authors declare that all the data supporting the findings of this study are available within the paper and its Supplementary Materials.

## Supplementary Materials

The following supporting information can be downloaded at: https://www.mdpi.com/article/10.3390/metabo16070471/s1.

## Abbreviations

The following abbreviations are used in this manuscript:

ELISA	Enzyme-Linked Immunosorbent Assay
ManNAc	N-acetylmannosamine
MTT	3-[4,5-dimethylthiazol-2-yl]-2,5-diphenyl tetrazolium bromide
OPLS-DA	orthogonal partial least squares discriminant analysis
PCA	principal component analysis
PDAC	pancreatic ductal adenocarcinoma
QCSP	quality control study pool
UHPLC-HRMS	ultra high-performance liquid chromatography–high-resolution mass spectrometry
VIP	variable importance in projection
