# Supplementary material for: Untargeted Metabolomics Analysis Reveals Potential Metabolic Targets in Gemcitabine-Treated Pancreatic Cancer Cells
Source: Metabolites. 2026 Jul 6;16(7):471. doi: 10.3390/metabo16070471 (PMC13413965; doi:10.3390/metabo16070471)
Supplement: Supplementary file 1 [file metabolites-16-00471-s001.zip › Supplemental Material.pdf]

Supporting information for

Untargeted Metabolomics Analysis Reveals Potential Metabolic Targets in Gemcitabine-Treated Pancreatic Cancer Cells

Arjun Prasad Tiwari [1,†], Blake R. Rushing [2,3,†], Larissa Silva [1], Susan J. Sumner [2,3,*] and Pinku Mukherjee [1,*]

[1] Department of Biological Sciences, University of North Carolina at Charlotte, Charlotte, NC 28223, USA;
atiwari5@charlotte.edu (A.P.T.); lsilva@charlotte.edu (L.S.)
[2] Department of Nutrition, UNC Chapel Hill, Kannapolis, NC 28010, USA,;
blake_rushing@unc.edu
[3] Nutrition Research Institute, UNC Chapel Hill, Kannapolis, NC 28010, USA
* Correspondence: susan_sumner@unc.edu (S.J.S.); pmukherj@charlotte.edu (P.M.)
† These authors contributed equally to this work.

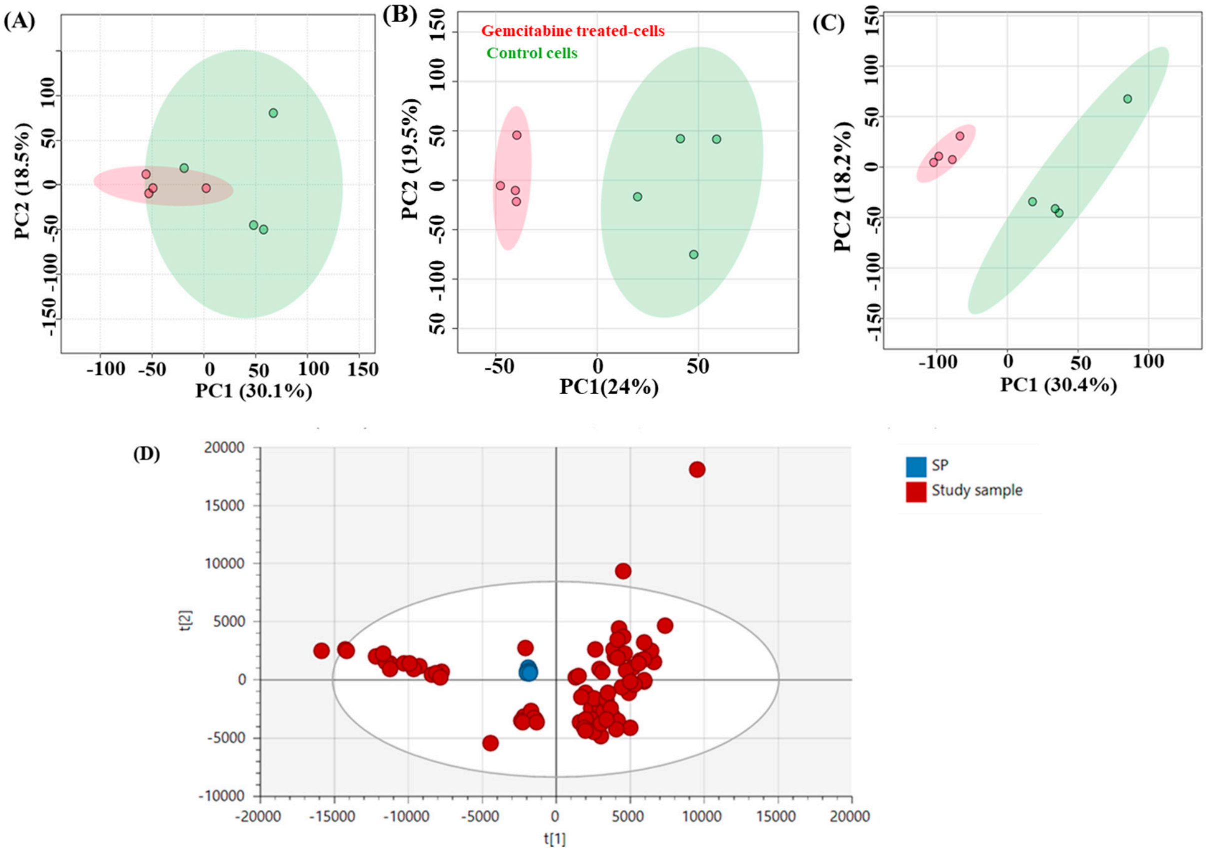

Supplemental Figure S1. *Principle component analysis (PCA) graph of metabolomics data for gemcitabine treated cells vs control cells; HPAF-II (A), MIA PaCa-2 (B) and BxPC-3 (C). (D) PCA of all study samples and study pools (SP) using all metabolomics peaks.*

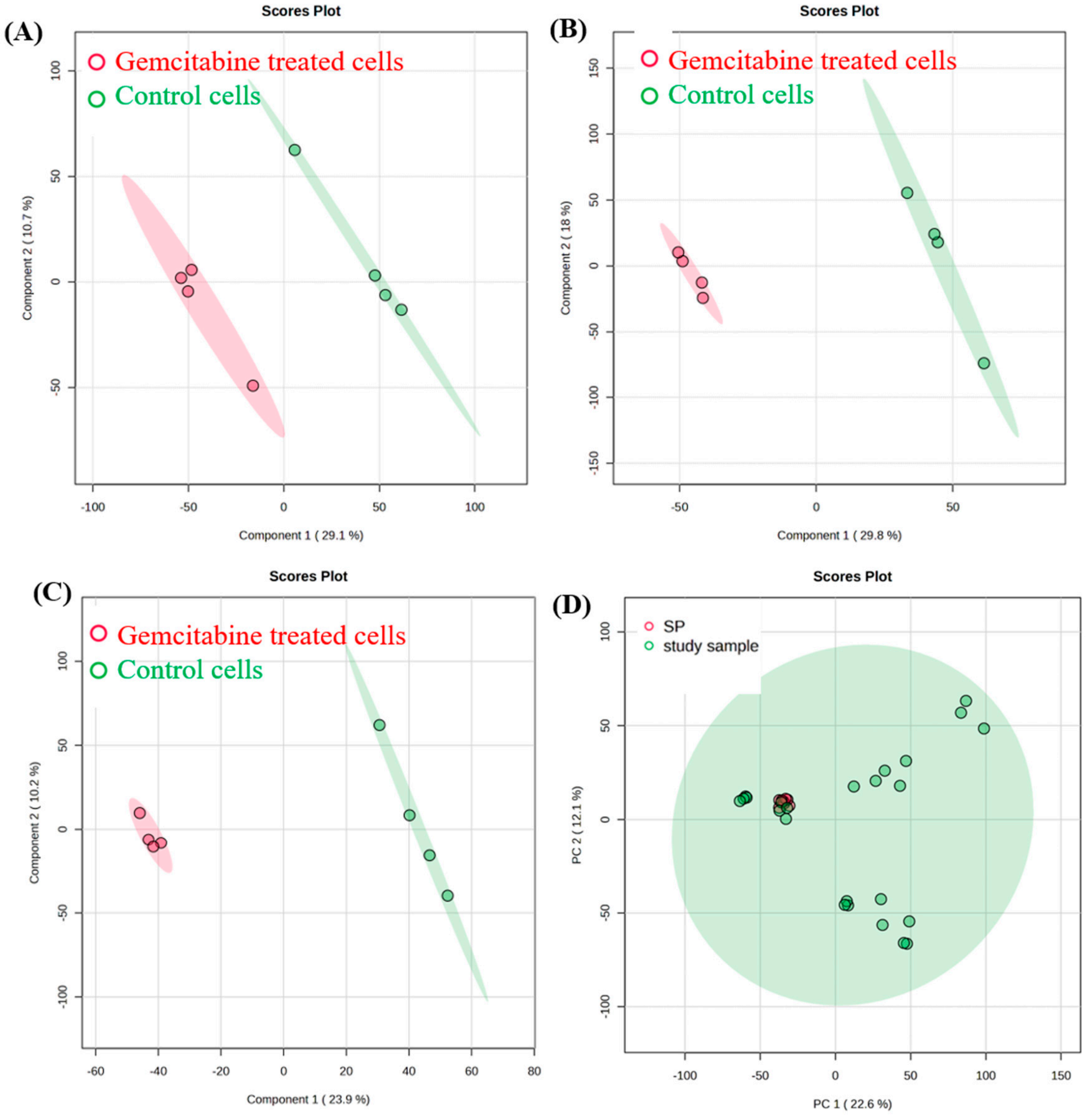

Supplemental Figure S2. *PLS-DA plots of gemcitabine treated cells vs control cells; HPAF-II (A), MIA PaCa-2 (B) and BxPC-3 (C). PCA plot of all study samples and study pools (SP) in the metabolomics dataset after filtering and normalization (D).*

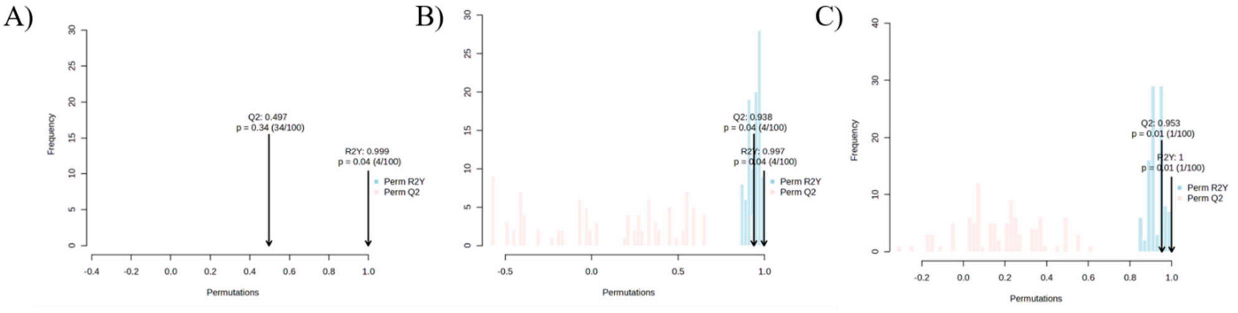

Supplemental Figure S3. *Permutation testing of OPLS-DA models for gemcitabine-treated pancreatic cancer cell lines. Permutation tests (n = 100 permutations) were performed to assess the robustness and predictive performance of the OPLS-DA models generated for HPAF-II, MIA PaCa-2, and BxPC-3 cells.*

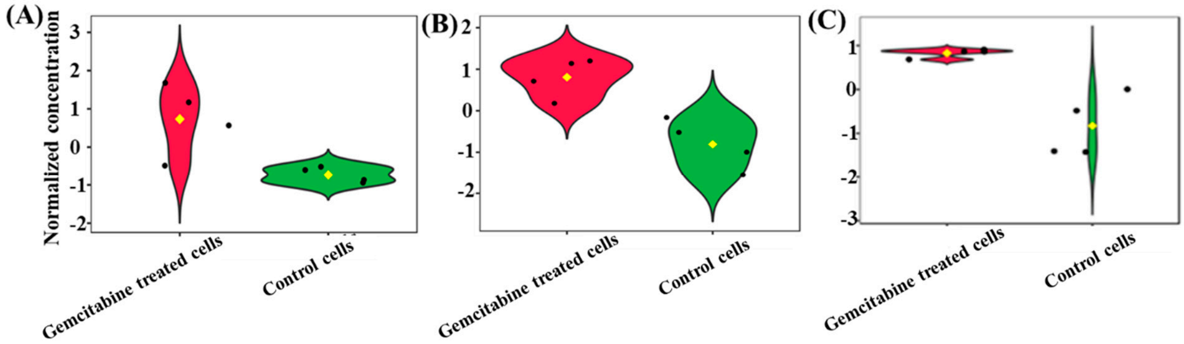

Supplemental Figure S4. *Normalized Glycerophosphocholine expression in different cells in gemcitabine treated and non-treated cells. A, B and C represent glycerophosphocholine level in HPAF-II, MIA PaCa2 and BxPC-3 cells respectively with p values 0.0216, 0.003 and 0.003, respectively. Data are displayed as auto scaled values.*

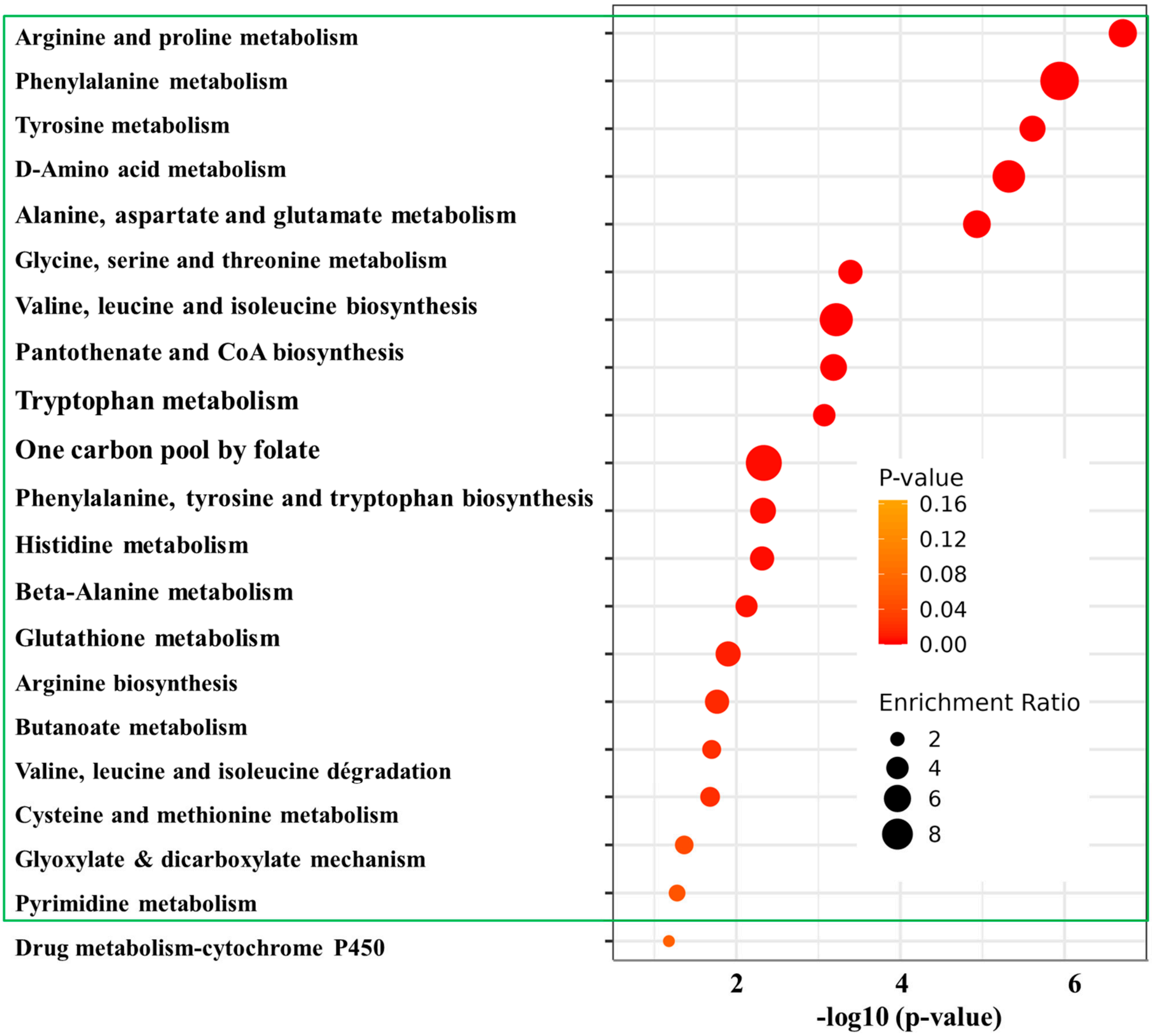

Supplemental Figure S5. *Pathway enrichment analysis of MIA PaCa2 cells. The pathways with p<0.05 are in a green box.*

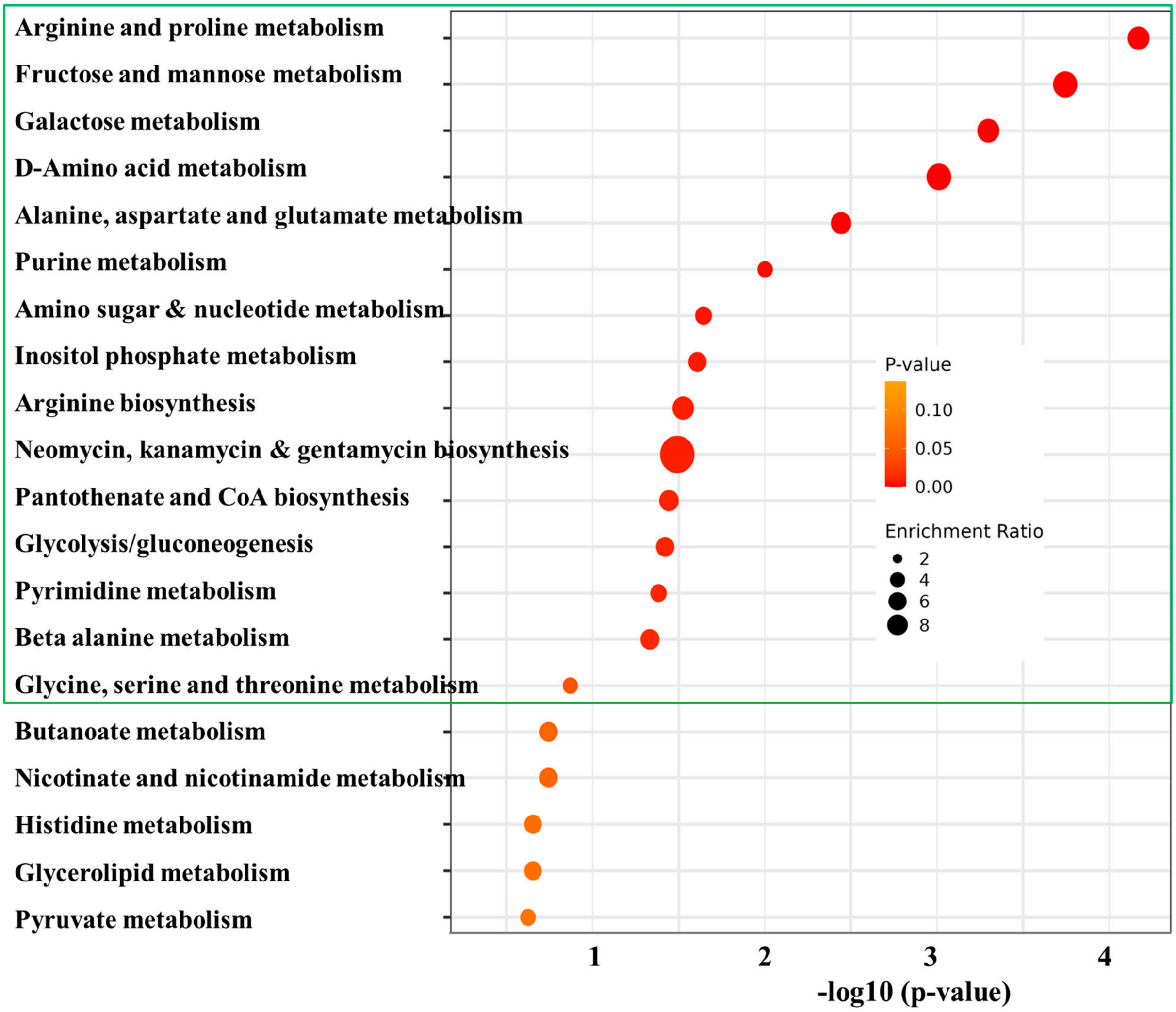

Supplemental Figure S6. *Pathway enrichment analysis of BxPC-3 cells. The pathways with p<0.05 are in a green box.*

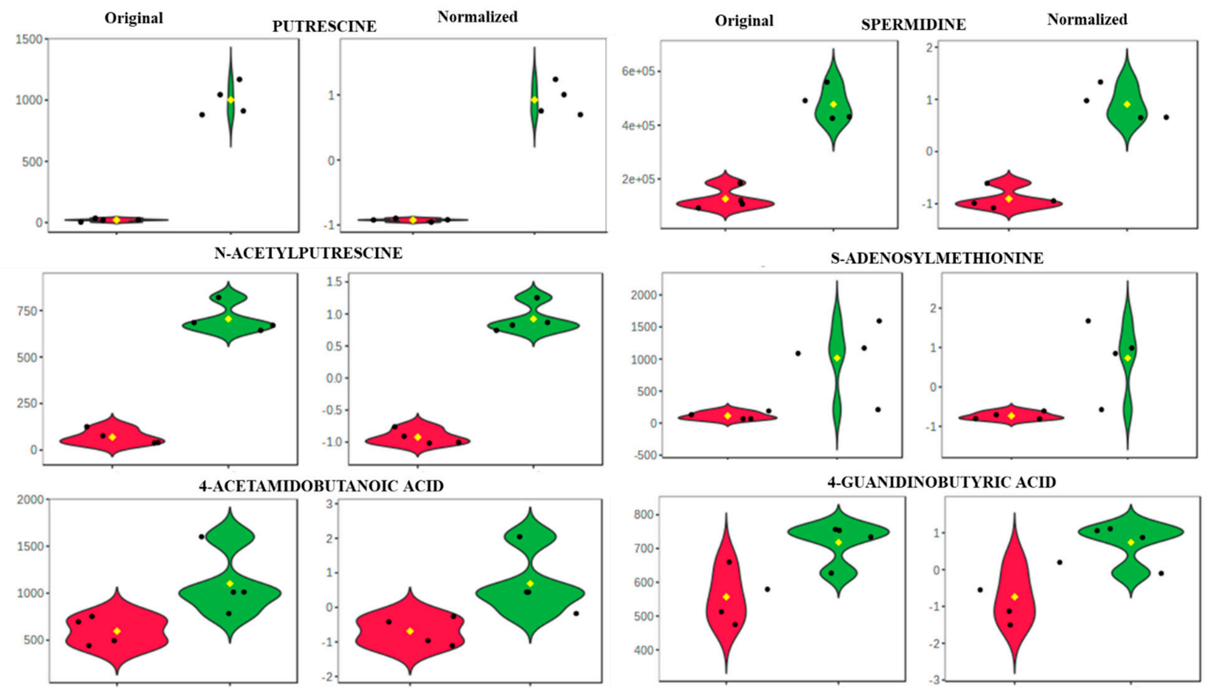

Supplemental Figure S7. *Metabolites associated with Arginine and proline metabolism in BxPC-3 cells. Putrescine (p=8.58E-05, spermidine p= 6.56E-04, N-acetyl putrescine p=7.07E-06, S-adenosylmethionine p= 2.14E-02, 4-acetamidobutanoic acid p=3.76E-02, and 4-guanidinobutyric acid p=1.93E-02). Data are displayed as peak areas (left) and as autsocaled values (right).*

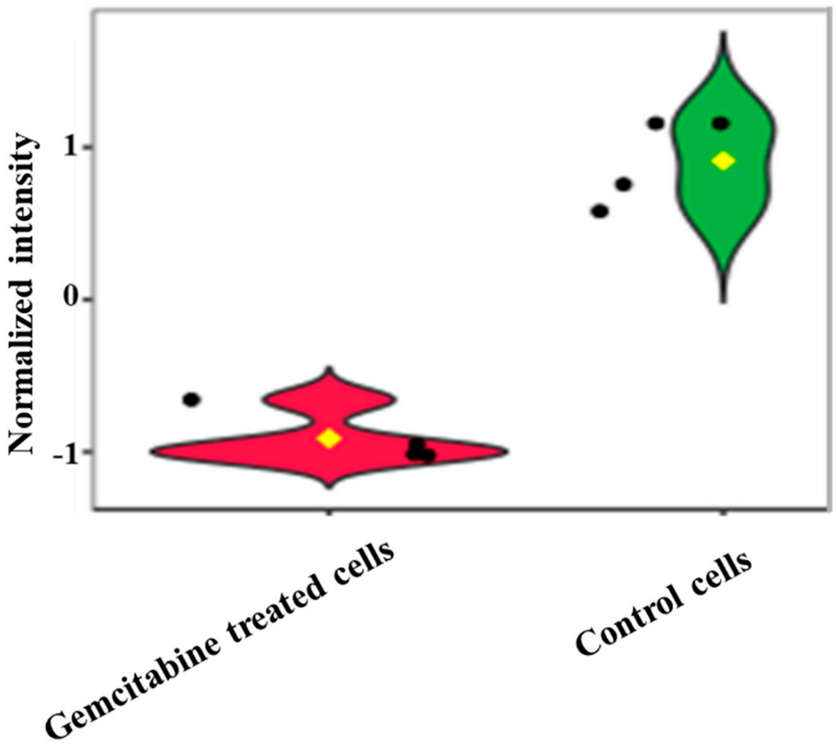

Supplemental Figure S8. *Normalized intensity of L-glutamine in BxPC-3 cells. Data are displayed as autoscaled values.*

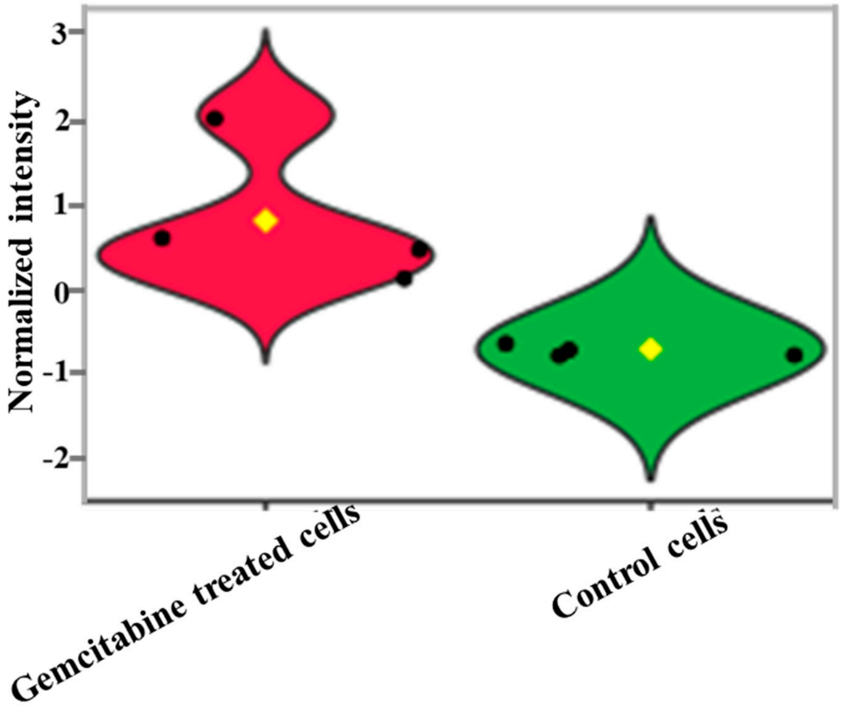

Supplemental Figure S9. *Normalized intensity of 7-dehydrocholesterol in HPAF-II (p=0.007). Data are displayed as autoscaled values.*

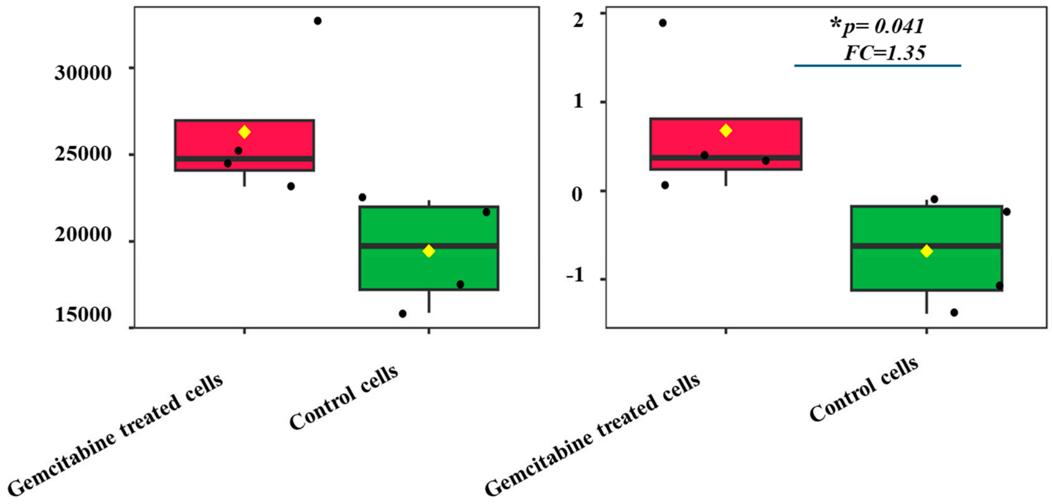

Supplemental Figure S10. *Peak area (left) and autoscaled intensity (right) of N-acetylneuraminic acid in untreated and gemcitabine treated MIA PaCa-2 cells.*

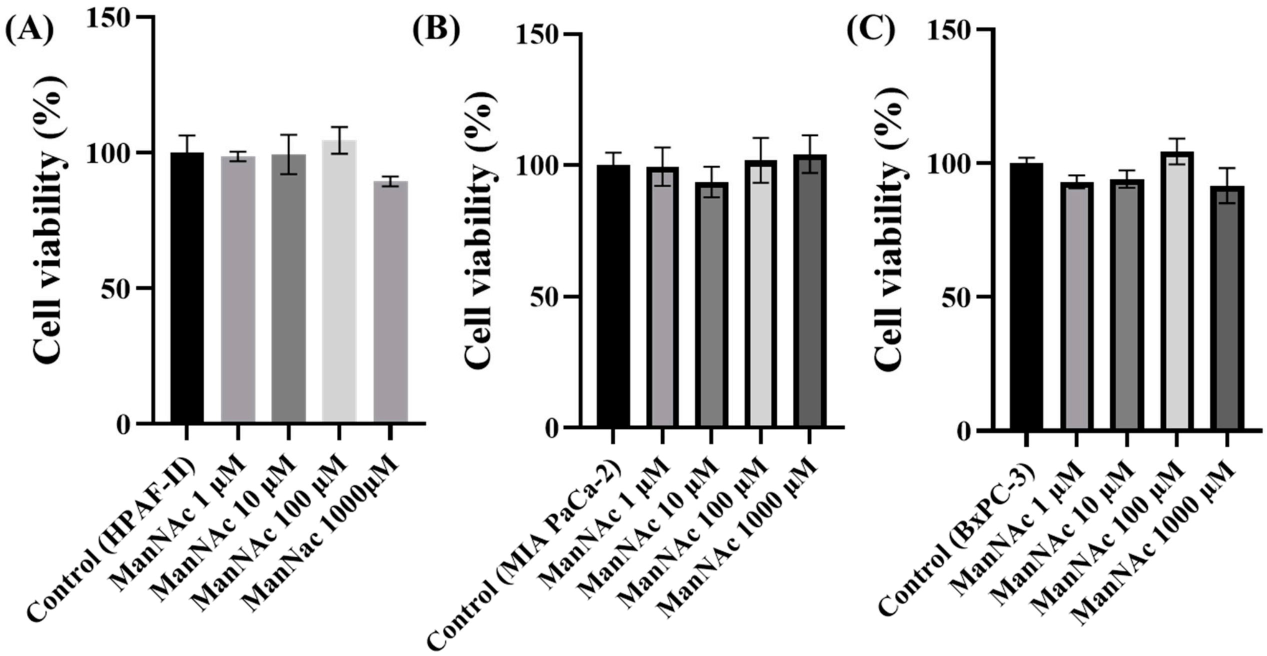

Supplemental Figure S11. *Cell viability assessment after ManNAc treatment for 48 h in HPAF-II (A), MIA PaCa-2 (B) and BxPC-3 Cells.*

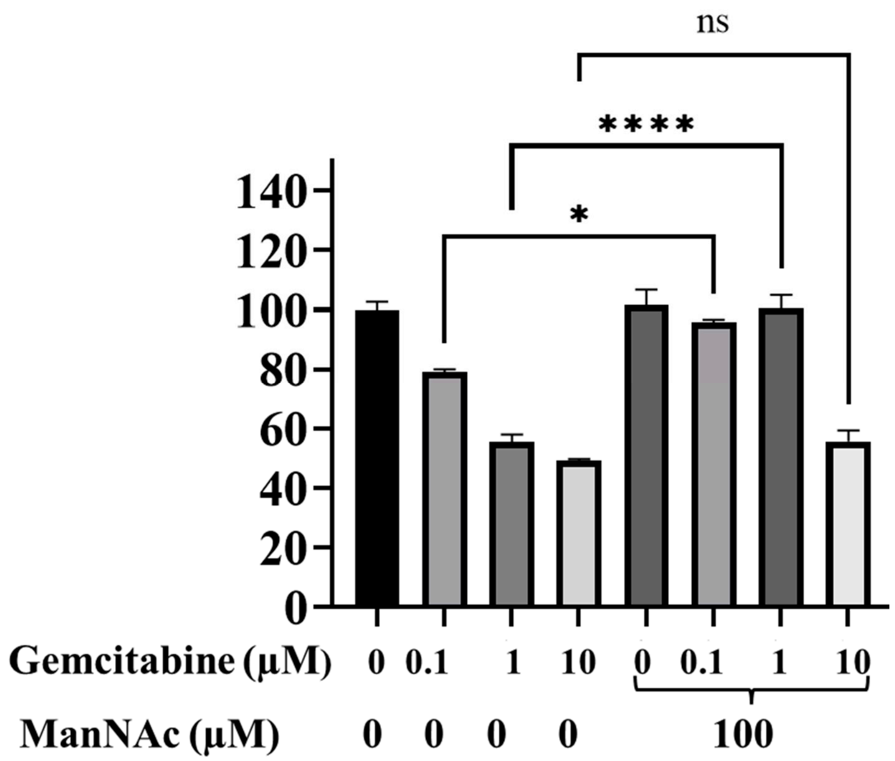

Supplemental Figure S12. *Cell viability assessment after co-treating gemcitabine/ManNAc for 48 h in MIA PaCa-2 cells. The p value <0.05 is considered significant. *, and **** represent p values <0.05, and <0.0001, respectively. Statistical significance was conducted by One-Way ANOVA (nonparametric or mixed model, multiple comparison, GraphPad).*

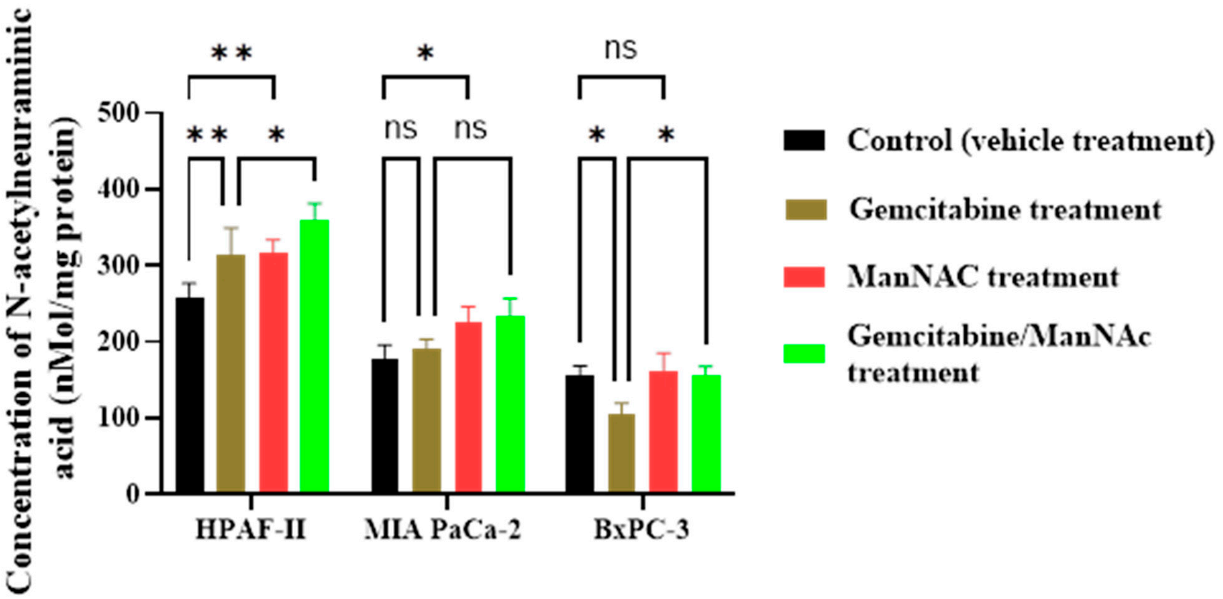

Supplemental Figure S13. *Effect of ManNAc supplementation on intracellular N-acetylneuraminic acid levels. Quantification of intracellular N-acetylneuraminic acid in HPAF-II, MiaPaCa-2, and BxPC-3 cells after treatment with gemcitabine, ManNAc, or gemcitabine plus ManNAc. Data are shown as mean ± SD (n = 3). Statistical significance is indicated as *P < 0.05 and **P < 0.01; ns, not significant. For this purpose, 10 µM, 1 µM and 0.1 µM gemcitabine, along with 100 µM ManNAc, were used for HPAF-II, Mia PaCa2 and BxPC-3 cells, respectively.*

Supplemental Table S1. *Model statistics (R2X, R2Y, Q2) for pair wise comparison.*

**Control vs Gemcitabine treated (HPAF-II)**	**p1**	**o1**
R2X	0.227	0.17
R2Y	0.837	0.161
Q2	0.537	0.0275
**Control vs Gemcitabine treated (MIA PaCa2)**	**p1**	**o1**
R2X	0.233	0.108
R2Y	0.981	0.0187
Q2	0.661	0.084
**Control vs Gemcitabine treated (BxPC-3)**	**p1**	**o1**
R2X	0.284	0.194
R2Y	0.972	0.0268
Q2	0.784	0.0474
**MIA Paca2 vs BxPC-3 (treated)**	**p1**	**o1**
R2X	0.616	0.108
R2Y	0.995	0.00434
Q2	0.981	0.00626
**HPAF-II vs BxPC-3 (treated)**	**p1**	**o1**
R2X	0.567	0.177
R2Y	0.976	0.0183
Q2	0.959	0.0127
**HPAF-II vs MIA PaCa2 (treated)**	**p1**	**o1**
R2X	0.467	0.135
R2Y	0.997	0.00242
Q2	0.955	0.0234
**MIA Paca2 vs BxPC-3 (Control)**	**p1**	**o1**
R2X	0.582	0.121
R2Y	0.982	0.0162
Q2	0.956	0.0197
**HPAF-II vs BxPC-3 (Control)**	**p1**	**o1**
R2X	0.536	0.11
R2Y	0.98	0.0191
Q2	0.954	0.015
**HPAF-II vs MIA PaCa2 (Control)**	**p1**	**o1**
R2X	0.518	0.13
R2Y	0.99	0.00977
Q2	0.951	0.00961
